# PSimScan: Algorithm and Utility for Fast Protein Similarity Search

**DOI:** 10.1371/journal.pone.0058505

**Published:** 2013-03-07

**Authors:** Anna Kaznadzey, Natalia Alexandrova, Vladimir Novichkov, Denis Kaznadzey

**Affiliations:** 1 Institute for Information Transmission Problems, Russian Academy of Sciences, Moscow, Russia; 2 Genome Designs, Inc., Walnut Creek, California, United States of America; 3 LabNext, Inc., Glenview, Illinois, United States of America; 4 DOE Joint Genome Institute, Walnut Creek, California, United States of America; Indiana University, United States of America

## Abstract

In the era of metagenomics and diagnostics sequencing, the importance of protein comparison methods of boosted performance cannot be overstated. Here we present PSimScan (Protein Similarity Scanner), a flexible open source protein similarity search tool which provides a significant gain in speed compared to BLASTP at the price of controlled sensitivity loss. The PSimScan algorithm introduces a number of novel performance optimization methods that can be further used by the community to improve the speed and lower hardware requirements of bioinformatics software. The optimization starts at the lookup table construction, then the initial lookup table–based hits are passed through a pipeline of filtering and aggregation routines of increasing computational complexity. The first step in this pipeline is a novel algorithm that builds and selects ‘similarity zones’ aggregated from neighboring matches on small arrays of adjacent diagonals. PSimScan performs 5 to 100 times faster than the standard NCBI BLASTP, depending on chosen parameters, and runs on commodity hardware. Its sensitivity and selectivity at the slowest settings are comparable to the NCBI BLASTP’s and decrease with the increase of speed, yet stay at the levels reasonable for many tasks. PSimScan is most advantageous when used on large collections of query sequences. Comparing the entire proteome of *Streptocuccus pneumoniae* (2,042 proteins) to the NCBI’s non-redundant protein database of 16,971,855 records takes 6.5 hours on a moderately powerful PC, while the same task with the NCBI BLASTP takes over 66 hours. We describe innovations in the PSimScan algorithm in considerable detail to encourage bioinformaticians to improve on the tool and to use the innovations in their own software development.

## Introduction

Sequence comparison is clearly the most ubiquitous task in bioinformatics. Standard tools for protein similarity search were established relatively early and did not change much over two decades. The growing demand for the analysis of ever growing datasets was satisfied by massive parallelization of public services, based mostly on NCBI BLASTP [1], [2], AB-Blast (formerly WU-Blast) [3] and FASTA [4]–[6] variations. This approach, however, posed certain difficulties for commercial sequencing projects, since building adequate private computing facilities was typically out of their scope, and caused recurrent emergence of commercial solutions based on specially tailored hardware, such as ones by CompuGen (compugen.com), TimeLogic (timelogic.com), Paracel (paracel.com, acquired and revived by Striking Development), and CLCbio (clcbio.com). In the past, hardware-accelerated solutions typically provided an advantage for a few years, after which they gradually retired due to being overgrown by the rapid performance progress of generic consumer platforms, so eventually pure software designs of search tools were brought back to competitive speed.

It is remarkable that, while quite an intense effort was aimed at the increase of search sensitivity (which led to the invention of many new tools and even concepts [2], [7]–[13]), for many years only a small number of studies were dedicated to the improvement of speed of the generic search [14]–[17]. In many cases, such works addressed particular problems and were actually not applicable to most generic search tasks [18]–[20]. In a sense, we could observe an ‘evolutionary conservation’ of early designs of similarity search methods, where the algorithm and even the implementation details stayed the same, while adaptation of the method to the changing requirements is done at a higher level by providing specific wrappers, pre- and post- processors [21]–[23], caching and reprocessing older search results [24], or running many instances of a tool in parallel [25]. Alternative homology detection methods [26]–[29] have also been developed, but they do not yet provide a real alternative to the classic alignment-based techniques.

In recent years, the emergence of Next Generation sequencing by 454, Complete Genomics, Illumina, Pacific Biosciences, Life Technologies (Ion Torrent), Applied Biosystems (SOLiD™) and Helicos BioSciences (tSMS™) spurred significant progress in the near-exact nucleotide sequence matching [30]. The advancement in sequencing technology provided previously unforeseen volumes of raw data [31], [32], including massive amounts of novel genome and metagenome sequences. Analysis of this data depends on the availability of quick and reliable methods of protein sequence similarity search, especially for tasks like ORF identification, primary annotation and functional prediction. With the growth of annotated sequence corpus, a two-pass strategy for these tasks becomes reasonable. In the first pass, close relatives of the proteins will be identified by a quick and relatively insensitive method. Then a more sensitive and slow search will be used only on the sequences lacking good hits. Rapid increase in the annotated protein diversity leads to reduction in the number of proteins analyzed during the second pass, making this strategy more efficient. To implement such strategy, a software tool capable of quick detection of strong similarities (possibly at a cost of reduced sensitivity) is required.

Recently, a few fast DNA similarity search tools were extended to allow for protein search. Those tools implement different speed enhancement algorithms. USEARCH [33] utilizes pre-sorting of the subject database using a similarity likelihood estimate based on the frequency of common k-tuples, where primary selection and sorting of candidates is based on the number of exactly matching k-tuples. Protein BLAT [18] allows for a single substitution in the matching k-tuple. Its suggested best usage scenario requires accumulation of multiple exact or near-exact k-tuple matches on a group of adjacent diagonals. RAPSearch [34], [35] uses a reduced alphabet which codes amino acid residues of similar properties with the same symbols. However, in our opinion, a generic fast protein similarity search tool suitable both for fragments and full-length proteins, while taking advantage of traditional gapped weight-matrix-based alignment scoring, is still missing.

Here we present a novel BLAST-like similarity search method that leads to a significant gain in performance compared to NCBI BLASTP, while maintaining reasonable sensitivity. We have implemented both nucleic acid and protein similarity search tools, which are available as parts of our QSimScan (Quick Similarity Scanner) software suite (available at SciDM.org). Due to optimization reasons, algorithm details for these variants significantly differ. In this article, we discuss the algorithm and its implementation for protein similarity search, and provide details on speed, sensitivity and selectivity depending on the search parameters and in comparison to SSEARCH, NCBI BLAST, USEARCH, BLAT and RAPSearch as a representative selection of tools utilizing different algorithmic approaches and also most commonly used for protein sequence search by the scientific community.

The PSimScan algorithm was developed for similarity-based annotation of massive volumes of genomic and metagenomic data, and optimized for a quick search of relatively strong similarities. When used for these tasks, it operates 5–100 times faster than NCBI BLASTP (which is still most commonly used for those tasks), and in a certain zone of parametric space delivers better recall to error ratios than any of the aforementioned fast protein similarity search tools. With parameter settings outside of the optimal zone, PSimScan can still be used for other tasks, such as weak remote homology detection or search for near-exact matches.

In this article, we will describe the PSimScan algorithm in detail sufficient for the open source community to improve on the tool and to reuse parts of the algorithm in modern bioinformatics software development to improve its performance and lower the hardware requirements. We will provide performance evaluation of our tool at different sets of parameters and compared to SSEARCH, BLAT, BLASTP, USEARCH and RAPSearch, however, detailed comparative evaluation of fast protein similarity search tools and their usage recommendations fall beyond the scope of this work.

## Algorithm Design

To gain maximum speed, similarity detection in PSimScan is organized as a pipeline of hit accumulators and filters connected in the order of increasing computational complexity. Initial hits are detected similarly to classic Pearson and Lipman’s reverse dictionary lookup [36]. Each subsequent step in the pipeline reduces the number of passed hits by either aggregating the neighboring ones or by dropping the weak ones, allowing to defer more accurate (and relatively heavy) computations to the later stages in the pipeline, when the majority of weak hit candidates are already sorted out.

First, we accumulate initial hits into ‘similarity zones’ aggregated from the neighboring matches on adjacent diagonals. We estimate similarity scores of such zones from the composition and placement of the initial hits. The similarity zones grow and merge as more initial hits are processed. After all initial hits have been seen, we detect and merge high-scoring zones which are properly arranged for a good alignment. Next, we compute optimal alignments on the merged zones and calculate statistical values for the high scoring ones. A pair of sequences may produce more than one alignment; these can optionally be filtered or merged into larger ‘super-alignments’.

Each stage of the algorithm is optimized for speed: in every case possible we are using direct addressing; all dynamic memory allocations are excluded by using an arena memory management pattern; moving objects in memory (including sorting) is avoided where possible.

**Figure 1:**
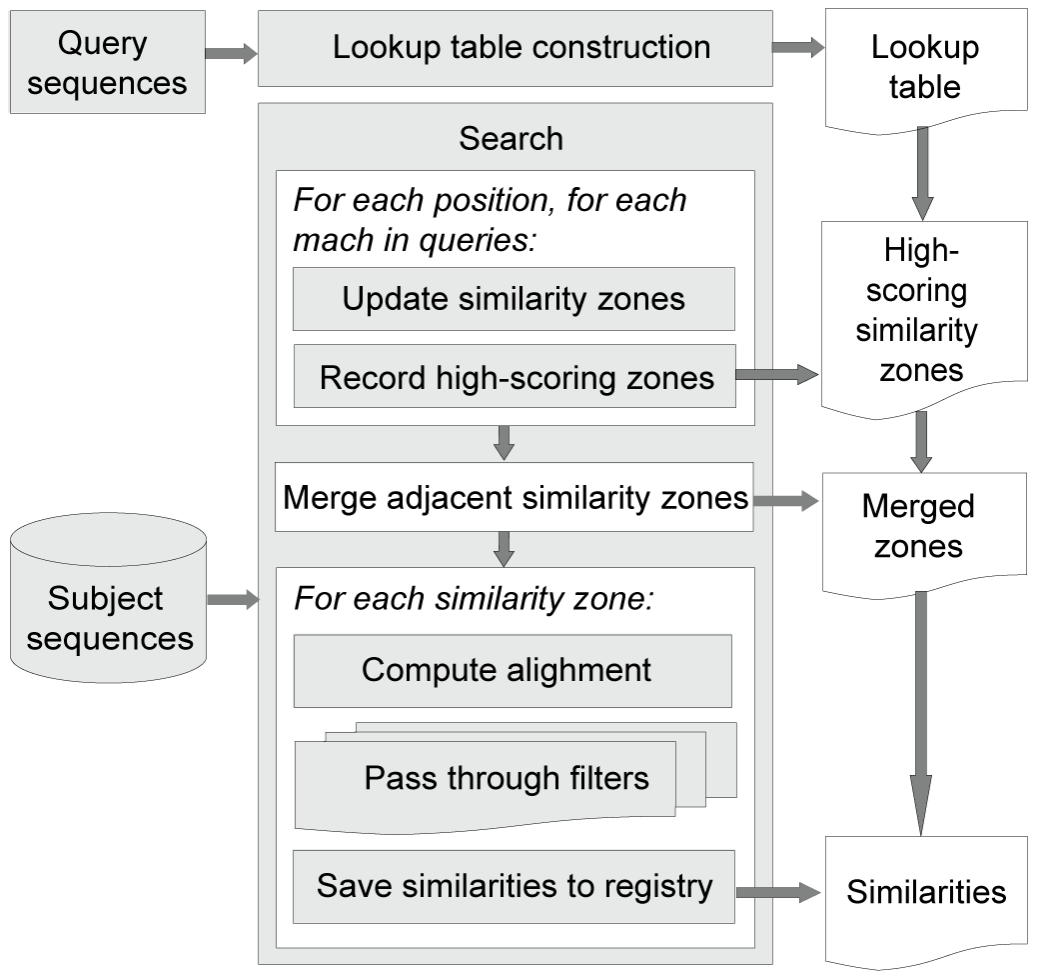
Block-scheme for PSimScan algorithm. Similarity detection in PSimScan is organized as a pipeline of hit accumulators and filters connected in the order of increasing computational complexity.

The outline of the PSimScan algorithm is presented on Fig. 1.

### Lookup Table Construction

The Lookup Table is a structure that associates each possible k-tuple with a list of locations in the query sequences where sufficiently similar k-tuples occur. The sufficient similarity is defined by scoring possibly similar k-tuples against those present in the query sequences using an amino acid substitution score matrix [37]–[40]. By default, BLOSUM62 is used; a different matrix may be specified as a search parameter. The similarity level is computed as the ratio of k-tuple-to-query match score to k-tuple’s self-match score. Only those locations on the query sequences where this ratio is above a certain ‘diversification threshold’ (a search parameter) are stored in the Lookup Table.

In the current implementation of PSimScan, we consider only ungapped matches of k-tuples to query sequences. Our testing showed that gapped matches add only a minute gain in sensitivity, but increase both computational and algorithmic complexity, thus increasing the search time.

In PSimScan, we build a lookup table on a set of query sequences prior to the actual search. This table is later used to retrieve locations of exact and inexact matches of k-tuples found in the subject sequences.

**Figure 2:**
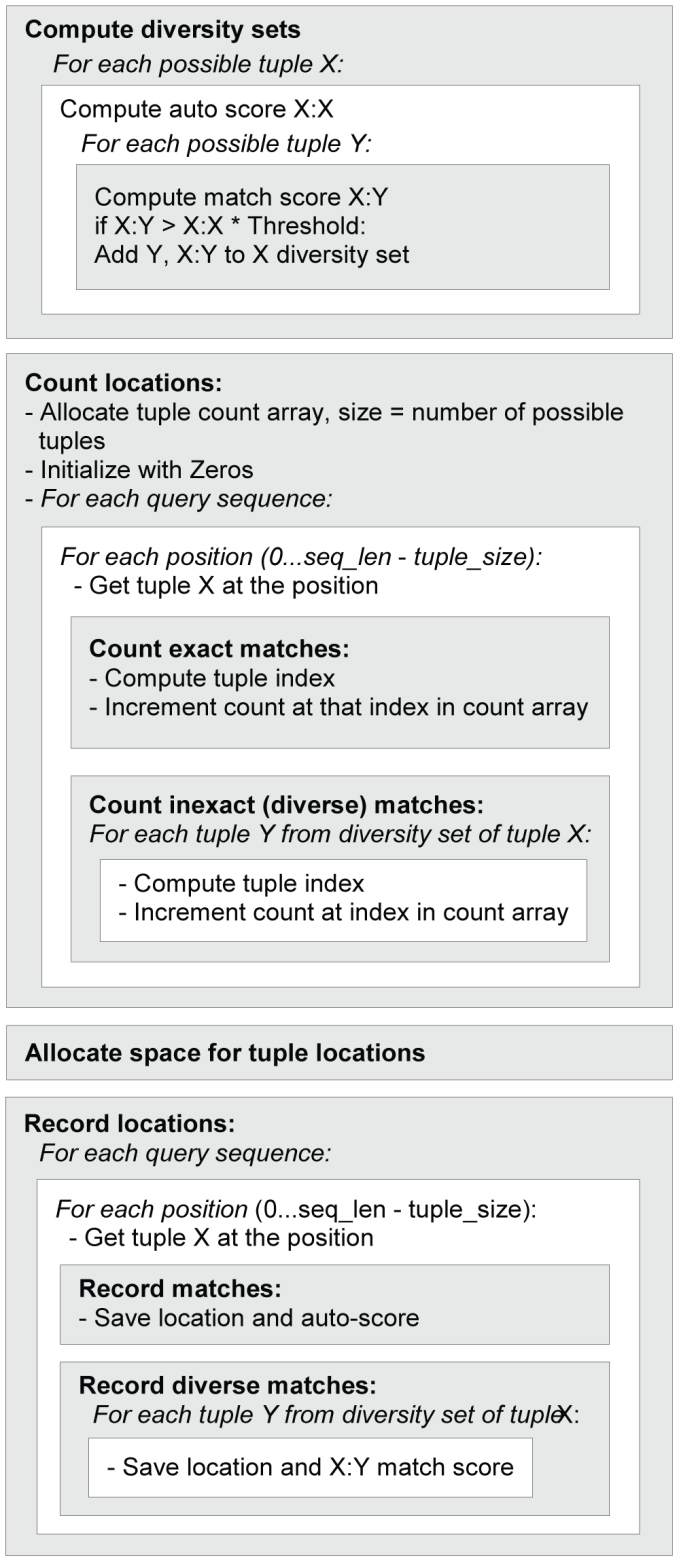
Block-scheme for dictionary construction. Location of every character tuple of the size K in the query sequences is recorded in a directly addressable Lookup Table, where a binary-converted tuple by itself serves as an index. Each entry in the table is a pointer to an array of locations.

Fig. 2 represents an outline of the Lookup Table construction. For each of the possible 20^k^ k-tuples, we record the locations in query sequences where ‘similar enough’ k-tuples are found. In a straightforward implementation, each of the possible 20^k^ k-tuples would be weighted for a similarity score against every k-tuple occurring in the query set, and the locations of those with sufficiently high similarities would be recorded. For the k-tuple size = 5, there are 3,200,000 (20^5^) possible variations; with a set of 4,000 query sequences of an average size of 300 amino acid residues (reasonable numbers for a single bacterial proteome) this translates into almost 4 trillion tuple-to-tuple comparisons. To reduce the amount of calculation and the time required for the lookup table construction, we use the following optimization. First, we record locations of all k-tuples present in the query sequences. Then, for each k-tuple occurring in the query set, we compute a list of k-tuples that match it with a sufficient score (the method of computing sufficient similarities will be described below). Then we update lists of locations of all these sufficiently similar k-tuples with the locations of the original k-tuple. Along with locations, we store the match scores between the original k-tuple and the sufficiently similar one. For the exact matches, this is a self-match score of the original k-tuple. These scores are used later at the search stage to estimate the strength of similarity zones composed from constituent k-tuple matches.

For computing k-tuples that are sufficiently similar to a given one, we use a recurrent procedure of *tuple diversification*. We assume that in the substitution matrix, a self-match score for any amino acid residue is the highest (this holds true for all NCBI-supplied matrices). First, we compute self-match scores for all prefixes and suffixes of a given k-tuple (T), and a score threshold S for the k-tuple, where S = (self match score)*(diversification threshold). Then we use a representation of all possible k-tuples as a 20-way k-level *trie* [41]. We recursively traverse this *trie* depth-first, computing the match score at each node. This score is defined as a sum of the score for the k-tuple prefix already seen (at prior recursion levels), the score of the substitution at the current position, and the score of the self-matching suffix of the original k-tuple. Once this score drops below S, we skip the entire sub-branch originating from that node. This is safe to do: according to our assumption that a self-match always scores the highest, no suffix can yield a match score greater than the self-matching one. When the recursion reaches its terminal leaves while the score stays above S, the k-tuple corresponding to that leaf is considered ‘sufficiently similar’ to the original and gets processed accordingly (see below). Such recursion traverses down the tree of all possible k-tuples, but the branches leading to a change beyond the diversification threshold are immediately terminated. This procedure efficiently enumerates all variants of k-tuples that are sufficiently similar to the original one.

To avoid dynamic resizing of k-tuple location lists (a costly procedure), the Lookup Table is computed in two passes over the set of query sequences. Beforehand, we allocate an array of TUPLE_INFO structures with the size equal to the number of all possible k-tuples (**A^k^**, where **A** is the alphabet size, 20 for the amino acid residues, and **k** is the k-tuple size). Each TUPLE_INFO structure holds a counter for the k-tuple occurrences, a pointer to the list of k-tuple locations, and an array of k-tuple’s partial (suffix) scores. A binary-converted k-tuple serves as an index into this array, so that the TUPLE_INFO structure associated with a given k-tuple is obtained in a constant time by direct addressing.

At the first pass over the query sequences, we count the exact matches for every k-tuple in the Lookup Table (every possible k-tuple). The tuple’s counters of occurrences are zeroed at the beginning. From each of the query sequences, we extract k-tuples one by one, starting at the position 0 and ending at (query length – tuple size), each time moving by one residue. For each extracted k-tuple, we increment the occurrence counter in its corresponding TUPLE_INFO structure. After the exact occurrences are counted, we *diversify* (as described above) each k-tuple that actually occurs in the query sequences (and thus has a non-zero occurrence counter). The diversification yields all k-tuples that are sufficiently similar to the original one. Then we add the number of occurrences of the original k-tuple to the counters of every ‘sufficiently similar’ k-tuple. Thus, at the end of the first pass, we have the count of the exact and close match locations for every k-tuple in the query set. Next, we sum up all these location counters and allocate a memory array sufficient to hold all these locations. The locations are held in the TUPLE_ENTRY structures (see below), so for the total N of locations we allocate an array of N TUPLE_ENTRYs. It is worth noting that since every location in the query sequences is recorded, N is always equal to (sum of the query lengths)–(k-tuple size)*(number of the queries). Then for each k-tuple, in its corresponding TUPLE_INFO structure, we record a pointer to the area within this array that will hold its locations. Such areas are spaced to allow holding exactly the counted (number of occurrences) TUPLE_ENTRY structures for each tuple.

Then we perform the second pass over the query set, now recording locations of the exact matches. At the end of the second pass, we diversify actually occurring tuples again, now physically copying locations of the matching tuples to the lists of locations for the sufficiently similar ones. We perform diversification again, because saving results of the first one would have caused more overhead then time saving. After the second pass is complete, we have a fully populated Lookup Table that operates in the following way: for each given tuple, its corresponding TUPLE_INFO structure is directly addressed in the *tuple_info* array at the offset equal to binary representation of that tuple. This structure holds a pointer to an array of tuple locations/match scores and an occurrence counter, which is equal to the size of the array. The array consists of TUPLE_ENTRY structures, each containing the query sequence index, the offset of the k-tuple in that query sequence and the array of suffix match scores (first suffix equals to the entire tuple match). The locations in the array are not ordered; the array contains blocks of exact locations of sufficiently similar tuples. The similarity search algorithm (see below) does not assume any particular order.

#### Initial match detection and weighting

**Figure 3:**
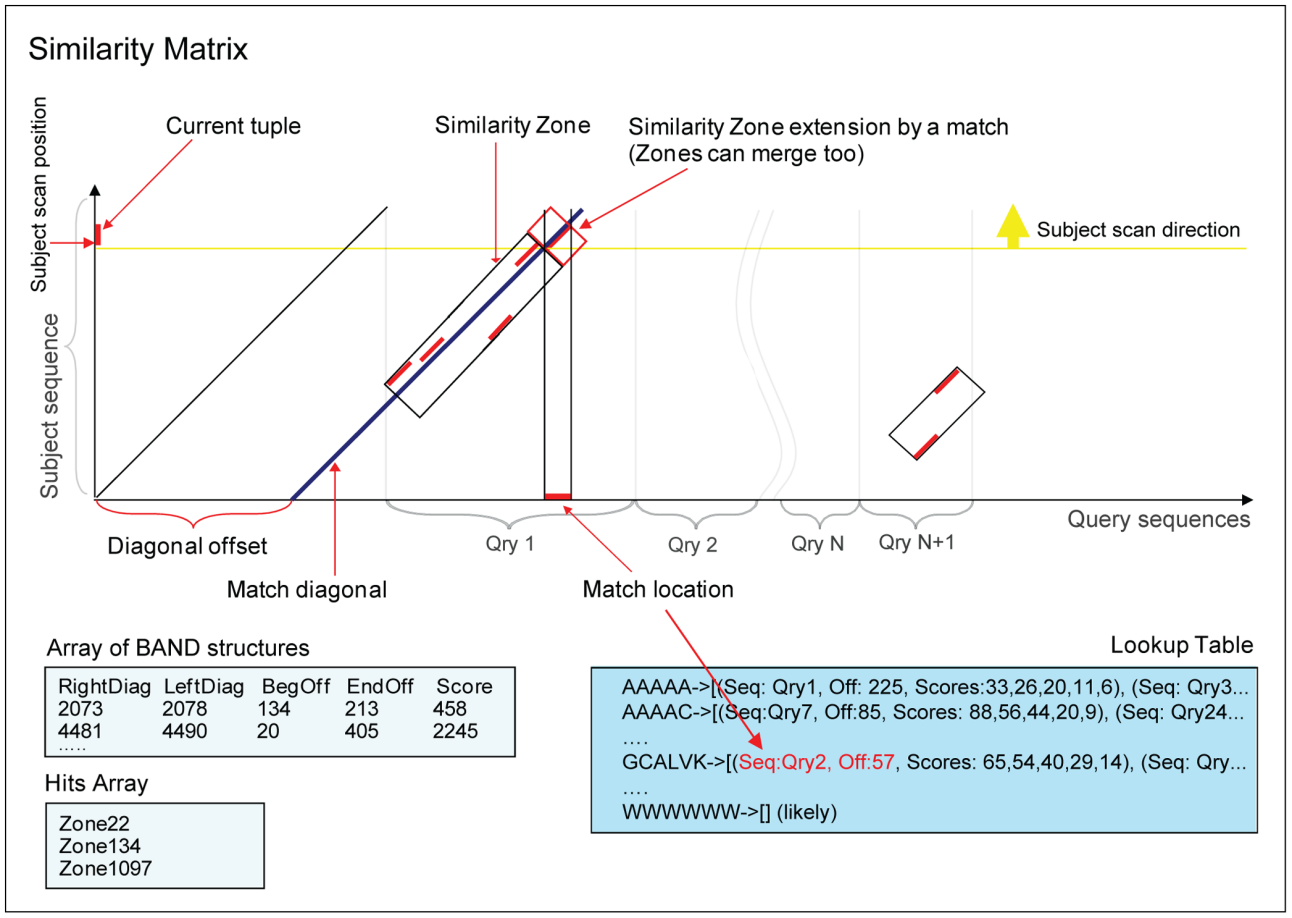
Main elements of the Search Space. ‘Similarity Matrix’ is a rectangular table with columns corresponding to positions in the query sequences and rows corresponding to positions in the ‘current’ subject sequence. Subject sequence database is processed record by record: k-tuples starting from every position are looked up in the Lookup Table, and locations of tuple matches (primary hits) are recorded. Neighboring primary hits form ‘Similarity Zones’, rectangular areas of Similarity Matrix oriented along the diagonals. Each new hit either joins an existing Similarity Zone that appears close by, or forms a new one. If such a hit appears close to more than one existing Zone, the two zones are merged. Similarity Zones are represented in PSimScan as BAND structures. Once a Similarity Zone’s score passes a *detection threshold*, it gets listed in the ‘Hits Array’.

The Similarity Matrix *is a* rectangular table with columns corresponding to positions in the query sequences, and rows corresponding to positions in the current subject sequence (Fig. 3). Each diagonal on the Similarity Matrix is represented by the *DIAGONAL_ENTRY* structure, which stores an offset of the last match on the diagonal, a score obtained by merging the last match with the diagonal’s *similarity zone*, a reference to the *similarity zone,* and an index of the subject sequence; the use of the latter will be described below. DIAGONAL_ENTRY structures are stored in the ‘*diagonals*’ array, where ‘diagonals’ offset in the Similarity Matrix serves as an index for the corresponding DIAGONAL_ENTRY structures.

S*imilarity zones* are rectangular areas on the Similarity Matrix, oriented along the diagonals and growing to include matches located in close proximity to each other. It cannot cross-sequence boundaries (see Fig. 3). S*imilarity zone* is represented by a *BAND* structure, which contains bounding coordinates, maximal cumulative match score achieved on this zone, and a query sequence index for this zone, used for tracking query sequence boundary crosses. Also, the BAND structure contains a ‘skip’ flag and a hit index (explained below).

Subject sequence database is processed record by record. For each subject sequence, a k-tuple that starts from every position is looked up in the Lookup Table (see above), and locations of its occurrences in the query sequences are retrieved. For every match we compute the offset of the diagonal where it lays. The corresponding DIAGONAL_ENTRY structure gets updated with the match location. The score of the associated similarity zone (see below) gets updated for the new match: the match score (minus the score of the prior k-tuple overlap) is added to the similarity zone score, while the score of any gap between the current and the closest prior match in the zone is subtracted.

The *match score* is a sum of weights (from a chosen substitution weight matrix) for all pairs of residues in the matched k-tuples. Match score calculation for sufficiently similar tuples is described above in the Lookup Table Construction section. To save time, all (full and partial) match scores between any k-tuples which are considered similar are computed once during the Lookup Table construction, and saved in the Lookup Table along with the k-tuple locations. Using these pre-computed partial (suffix) match scores allows performing zone score update for partially overlapping matches fairly quickly, without accessing substitution weight matrix for every matched residue pair.

**Figure 4:**
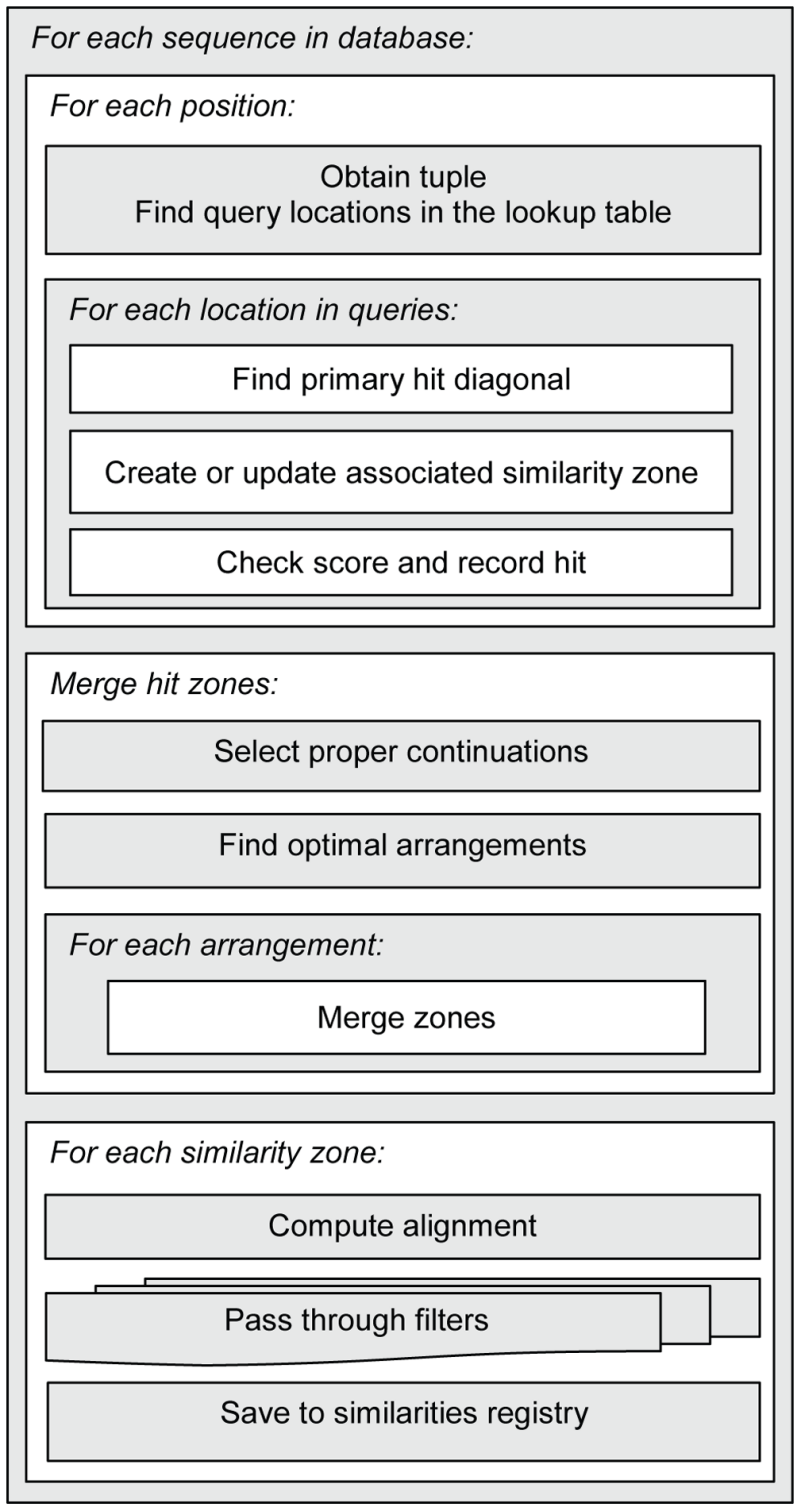
Block-scheme for the inner search loop. For each subject sequence, k-tuples starting from every position are looked up, and the match locations are recorded. The offset of the diagonal where the match appears is computed, and the associated diagonal control structure is updated with the location and the score of the match. On the accumulation stage, multiple neighboring primary hits are aggregated into Similarity Zones, reducing the number of items for processing. Such zones, in turn, may merge into a smaller number of larger aggregates.

An overview scheme of the search loop is presented on Fig. 4.

The total number of diagonals in the Similarity Matrix equals to (sum of query sequence lengths)+(subject sequence length). The lengths of the query sequences are known at the very start of a search, while subjects can be arbitrary. To exclude dynamic memory management overhead, we pre-allocate an array of DIAGONAL_ENTRY structures for the pre-defined maximal subject length, which is a search parameter (with default value of 50,000).

#### Similarity zone detection

On the accumulation stage, multiple neighboring primary hits are aggregated into *similarity zones*, thus reducing the number of items for processing. Such zones, in turn, may merge into a smaller number of aggregates. Only zones collecting critical cumulative scores of primary hits can trigger further processing.

Each diagonal at any given time is associated with only one similarity zone. The algorithm ensures that if a second distinct similarity zone appears on the same diagonal, its detection starts only after the previous similarity zone is completely processed: this is implied by the ordered retrieval of the k-tuples from the subject sequence.

When a primary match occurs on the diagonal, the corresponding DIAGONAL_ENTRY structure can be found in one of the following states: 1) unused; 2) used with an earlier subject sequence; 3) used with a different query sequence and 4) already used with the same query/subject sequence pair. The first two cases occur when the first match for the present subject sequence is detected on the diagonal. A new similarity zone enclosing this match is then created, and a reference to this zone is recorded in the DIAGONAL_ENTRY. The third case is treated similarly to the first two. The DIAGONAL_ENTRY is re-used with a new query sequence. It is safe, since the algorithm ensures that any previously found high-scoring similarity zones intersecting this diagonal had been already saved.

In the fourth case, a new match is found on the diagonal that already intersects with some similarity zone on the same query/subject pair. This new match can be a fair continuation of an existing similarity zone, or it can reside too far apart. To distinguish these possibilities, the extended zone score is estimated by adding the original zone’s score to the new match’s score, and subtracting a penalty for the gap. We used the affine gap penalty calculation method with pre-defined gap initiation and extension penalties (search parameters) [42]. When a new match overlaps a similarity zone, the score is adjusted so that the overlapping portion is not counted twice. If the score estimate is positive, the similarity zone is extended to include the new match. If it drops below zero, a new similarity zone is allocated for the diagonal and initialized with the match score and location. In the former case, the similarity zone previously attached to the diagonal also remains ‘active’; with further processing it can potentially be extended by more matches or merged with other zones.

After a match has been processed, the adjacent diagonals are searched for similarity zones that can be merged with the current one. No more than *mxshift* diagonals are looked up in both directions (where *mxshift* is a search parameter). For each possible merge a score is estimated. Those that score positively get merged; all involved diagonal control structures are updated to refer to the resulting zone. After two zones merge, one of them expands to include the area previously covered by both similarity zones, and another is marked ‘obsolete’ by raising the ‘skip’ flag. This flag is also used later when processing high scoring zones.

In order to avoid the overhead of dynamic memory management, we pre-allocate an array of BAND structures, reasonably large to be enough for practical purposes. By default, we allocate 2 million BANDs; this number can be changed at the compiling time. We keep a counter of used similarity zones, which serves as an index of the first BAND structure yet unused. This counter is zeroed at the beginning of each subject sequence processing. When a new similarity zone needs to be initiated, the BAND structure at this index is taken, the ‘skip’ flag in it gets reset, and the counter is incremented (also, the hit index of the similarity zone is set to a sentinel value; the use of hit index is discussed below). If the counter reaches its pre-allocated limit, no further zones are recorded, and the program issues a warning.

#### Similarity zone processing

Once an updated similarity zone score passes a detection threshold, it gets recorded in the ‘hits’ array. After an entire subject sequence is processed, all such zones will be referenced in the hits array, even if their scores have later dropped. Each element of the hits array is an index of the high scoring BANDs. The same method as for the BANDs array is used to avoid dynamic re-allocation of the hits array: a reasonably large number of hit elements are pre-allocated and the ‘fill’ counter is used as an index of first unused element. At the beginning of subject sequence processing, this counter is zeroed. Whenever a similarity zone passes the score threshold, it is checked for being already referenced in the hits array: for already referenced zones the ‘hit index’ holds a non-sentinel value. If it is not yet referenced, the index of this zone in the BANDs array is recorded in the *‘fill counter’* element of the hits array, the *fill counter* value is recorded as the zone’s hit index, and the *fill counter* gets incremented by one. In the rare cases when the hits array is exhausted, the hits accumulation stops and the program issues a warning.

After all k-tuples from the subject sequence are processed, the hits array is used to enumerate similarity zones with high scores. Some of these zones could be already merged into larger ones; such zones have their ‘skip’ flag set, and they are ignored. The remaining high-scoring zones are regularly built around narrow diagonal bundles, limited by the extent of lateral search for adjacent zones to merge (*mxshift* parameter). The complexity of our similarity zone detection algorithm grows linearly with the number of adjacent diagonals being checked; therefore, checking more than a few (2–5) is impractical. In order to bring together zones separated by longer gaps, we perform a single linkage clustering on the entire set of valid zones, using pairs of ‘compatible’ zones as links. The zones are considered compatible if the sum of their scores, adjusted for the non-overlapping fractions, is larger than the gap penalty computed on a minimal distance (in diagonals) between these zones. The clusters are computed using a very efficient original transitive closure algorithm (please see the source code for details). Then the contents of each cluster are merged into the first zone in that cluster; the remaining zones are marked obsolete.

The boundaries of each resulting composite similarity zone mark out a diagonal band on the *similarity matrix*, which contains all primary k-tuple matches (Fig. 3). Complete *similarity zones* are extended in all directions by a certain user-defined factor, and then processed using a ‘banded’ optimal alignment method based on Needleman-Wunch algorithm [43]. Obtained optimal alignments are passed on to the subsequent processing stages. While performing alignment, the Smith-Waterman scores [44] are computed.

#### Filtering and post-processing

A number of optional similarity filters are available. The most trivial one is the *alignment score filter* allowing for sorting out similarities which score below a certain threshold. The filter is applied after the accurate Smith-Waterman scores are computed, therefore this stage of filtering is more reliable than the ones used at any of the previous steps.

The *long repeat* filter detects when multiple similarities between a pair of sequences are caused by repeats in one or the both of them. It would drop all such similarities from the results, except for the best scoring ones.

The *remote batch merger* is capable of merging remote similarity zones separated by non-similar zones or long gaps. By nature of the PSimScan algorithm, it detects only compact similarity zones, so if there are two pairs of similar domains separated by a long non-similar zone, PSimScan will report two short similarities. In certain cases it is convenient to report such cases as one similarity, even though it contains either a gap that would yield too big an affine penalty or a non-similar zone where the true Smith-Waterman score would drop to zero. The remote batch merger uses a dynamic programming-based algorithm in order to find the best arrangement for the aligned segments.

After local alignments are built and remote similarity zones are merged, the Karlin-Altschul statistics [45] are computed on the merged alignments, yielding standard E-value scores, to assess and compare qualities of found similarities. We used the ‘ariadna’ library from WTCHG [46] for an accurate estimate of the gapped alignment score. The E-values produced with it match those computed by the SSEARCH [4] tool. It is also worth noting that while those E-values are very similar to the ones reported by USEARCH [33], they differ from those reported by NCBI BLAST. For a detailed assessment of the quality of different E-value computing methods see Brenner *et al* [47].

To summarize, instead of building alignments along individual diagonals as traditional BLAST-like algorithms do, PSimScan grows similarity zones across arrays of diagonals, consecutively filtering out diagonals with no hits, low-scoring similarity zones and hit arrays, allowing for a higher computational efficiency.

## Methods

The algorithm described above is used as a core of PSimScan, an open source software package for protein similarity search available under GPL license. The software is implemented in ANSI-compliant C++ compatible with GCC compilers version 3 and higher and Microsoft compilers version 12 and higher. The Makefiles for Gnu environment are available. PSimScan has been tested on Linux-32 and Linux-64 systems and on Windows XP and Vista. The source code and Makefiles are available at code.google.com/qsimscan.

### Input and Output Formats

PSimScan takes FASTA-formatted sequence files as input and writes results to flat files. Similarities can be reported in one of the following formats:

- PSIMSCAN TEXT, similar to NCBI BLAST TEXT, which shows alignments and verbose information on the hits. The format details and the amount of the output data can be controlled by user defined parameters;
- TABULAR/EXTENDED TABULAR, one tab delimited line per similarity;
- NCBI m8/m9.

### Control Parameters

The application is controlled via command line or configuration file. A complete list of parameters, their default values and explanations, can be obtained by running the software in ‘help’ mode, with the ‘-h’ command line switch. Each entry in the configuration file corresponds to a command line option; the command line takes precedence over the configuration file.

The most important parameters are:

–ksize: k-tuple (dictionary word) size
–approx: tuple diversification level as a fraction of the original k-tuple’s self-match score
–kthresh: similarity zone detection threshold, a minimal zone score triggering further processing
–omode: output format, from one of these: TEXT, TAB, TABX, NCBI_M8, NCBI_M9
–*rpq*: number of results per query to keep and report.

## Results

To assess performance characteristics of PSimScan, we used two types of tests. First, we evaluated the quality of similarity detection by PSimScan using the ‘golden set’ of similarity-independent homologies, as described by Brenner *et al* [47]. Second, we have compared the performance of the PSimScan to other industry standard tools in a real-life scenario: a comparison of an entire bacterial proteome to a standard protein database.

### Test Arrangement

The following sequence sets were used:

PDB90 subset of the Astral SCOP database ver.1.75, containing 15545 sequences of the total length of 2683934 amino acid residues, classified into 3901 unique folds.

*Streptococcus pneumoniae* R6 proteome, downloaded from NCBI ftp site (at ftp://ftp.ncbi.nih.gov/genomes/Bacteria/Streptococcus_pneumoniae _R6/NC_003098.faa) in April 2012, which contains 2,042 protein sequences with the total length of 588, 745 amino acid residues.

NCBI non-redundant protein database (“nr”) of 16,971,855 proteins, downloaded from NCBI ftp site (at ftp://ftp.ncbi.nih.gov/blast/db/fasta/nr.gz) in April 2012, which contains 5,870,692,704 amino acid residues, of total size of 9.47 Gb,

SwissProt/Uniprot database of 534,695 proteins, downloaded from NCBI ftp site (at ftp://ftp.ncbi.nih.gov/blast/db/fasta/swissprot.gz) in April 2012, which contains 189,663,123 amino acid residues, of total size of 254 Mb,

The BLOSUM62 [40] substitution weight matrix was used.

Testing was performed on the following computer system:

AMD Athlon(tm) II X2 215 (2700 MHz)-based desktop computer with 8 GB DDR2 RAM (800 MHz) and NFS-mounted disk storage (over a Gigabit Ethernet network), running Red Hat Fedora 16 Linux OS (64-bit).

PSimScan executable was compiled locally from source code (revision 49) using gcc++ compiler version 4.6.3 with optimization level −O2.

The comparison was done with SSEARCH version 36.3.5a (locally compiled from source code), NCBI BLAST version 2.2.25 (64-bit binary), RAPSearch version 2.04 (64 bit, locally compiled from source code), USEARCH version 5.1.221 (32-bit binary, the only version available for free non-commercial use), and BLAT version 34.12 (64-bit binary).

#### Sensitivity/selectivity assessment with PDB90 benchmark

**Figure 5:**
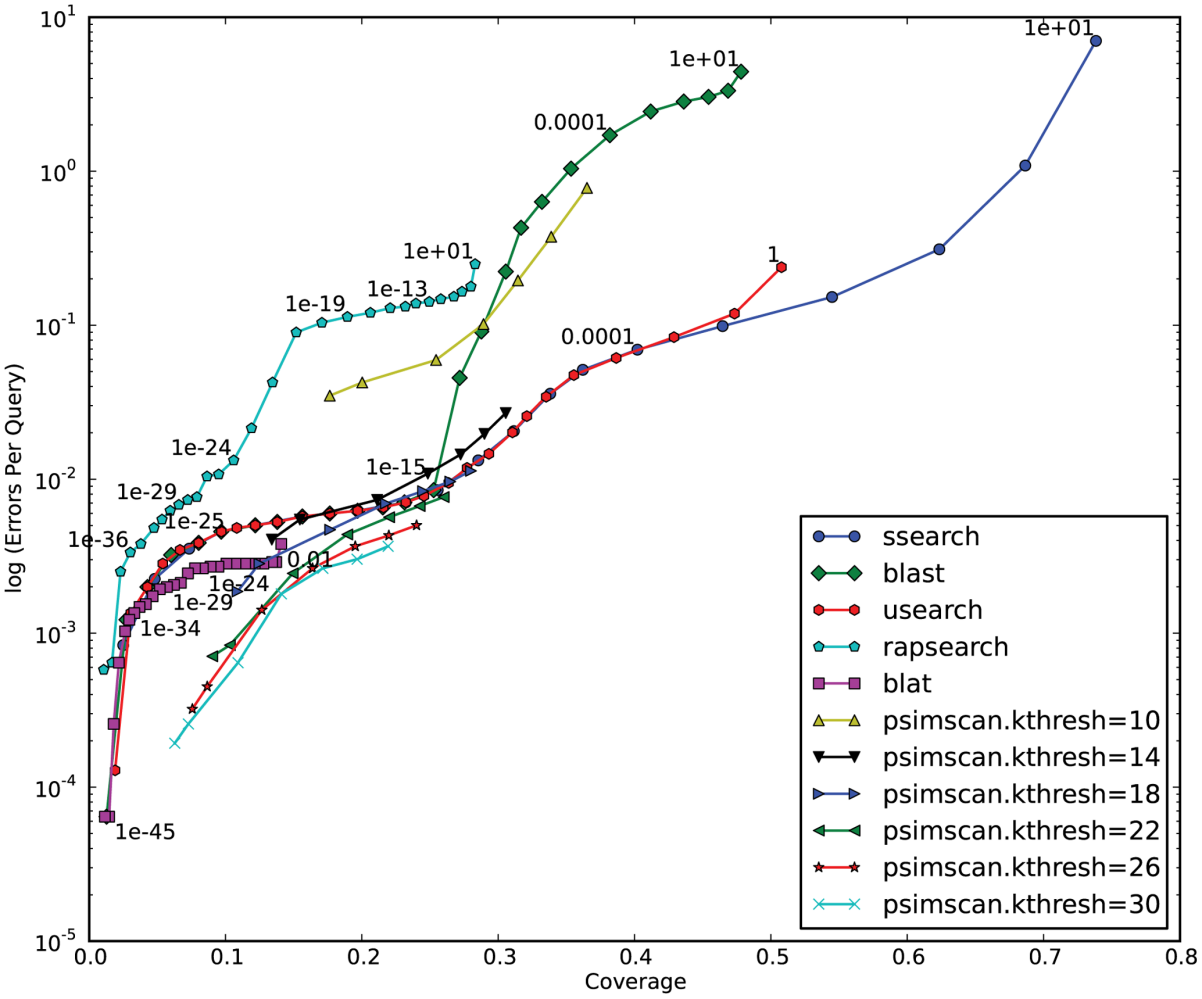
Selectivity and Sensitivity of PSimScan at different parameters versus other similarity search tools. All of the proteins in the PDB90 database were compared with each other using PSimScan, SSEARCH, BLAST, USEARCH, RAPSearch and BLAT. PSimScan was tested at different combinations of *kthresh* (similarity zone detection threshold) and *approx* (tuple diversification level) parameters. For SSEARCH, BLAST, USEARCH, RAPSearch and BLAT, the Coverage vs Error graphs were plotted as described by Brenner *et al* [47]. Similarities between proteins of the same SCOP fold were treated as true positives, while similarities between proteins of different folds – as false positives (errors). The Coverage is the ratio between the number of true positives and the total number of protein pairs, where both members belong to the same fold. The EPQ is the ratio between the number of detected false positives and the number of queries. The Coverage-vs.-Error graph contains points in the Coverage/EPQ plane which correspond to the sets of similarities with E-values below a given cut-off (some dots on the graphs are labeled with E-values). To get comparable graphs for different tools, we re-computed the E-values for all detected similarities with SSEARCH, and used those E-values for the graph construction. We ran PSimScan at all combinations of 6 different *kthresh* values (shown in legend) and 7 different *approx* values. For each run, total coverage and EPQ were computed and plotted. On each curve corresponding to a particular *kthresh*, the triangles mark the following *approx* values, left to right: 1.0, 0.95, 0.9, 0.85, 0.8, 0.76, 0.72.

We used the PDB-90 subset of Astral database [48] as the test set. PDB90 contains sequences of solved protein structures that have been manually classified into superfamilies and folds. Each sequence from the database was used as a query to search the database. We counted similarities between proteins of the same fold as true positives, and similarities between proteins of difference folds as false positives. Based on these counts and following the protocol described by Brenner et.al. [47] we computed EPQ (Errors Per Query) and Coverage (fraction of total positives detected) values, and plotted them for the runs of PSimScan at different parameters settings. For comparison, we outlined in the same coordinates the coverage-vs.-error plots for five most commonly used protein similarity search tools ran on the same data (Fig. 5).

**Figure 6:**
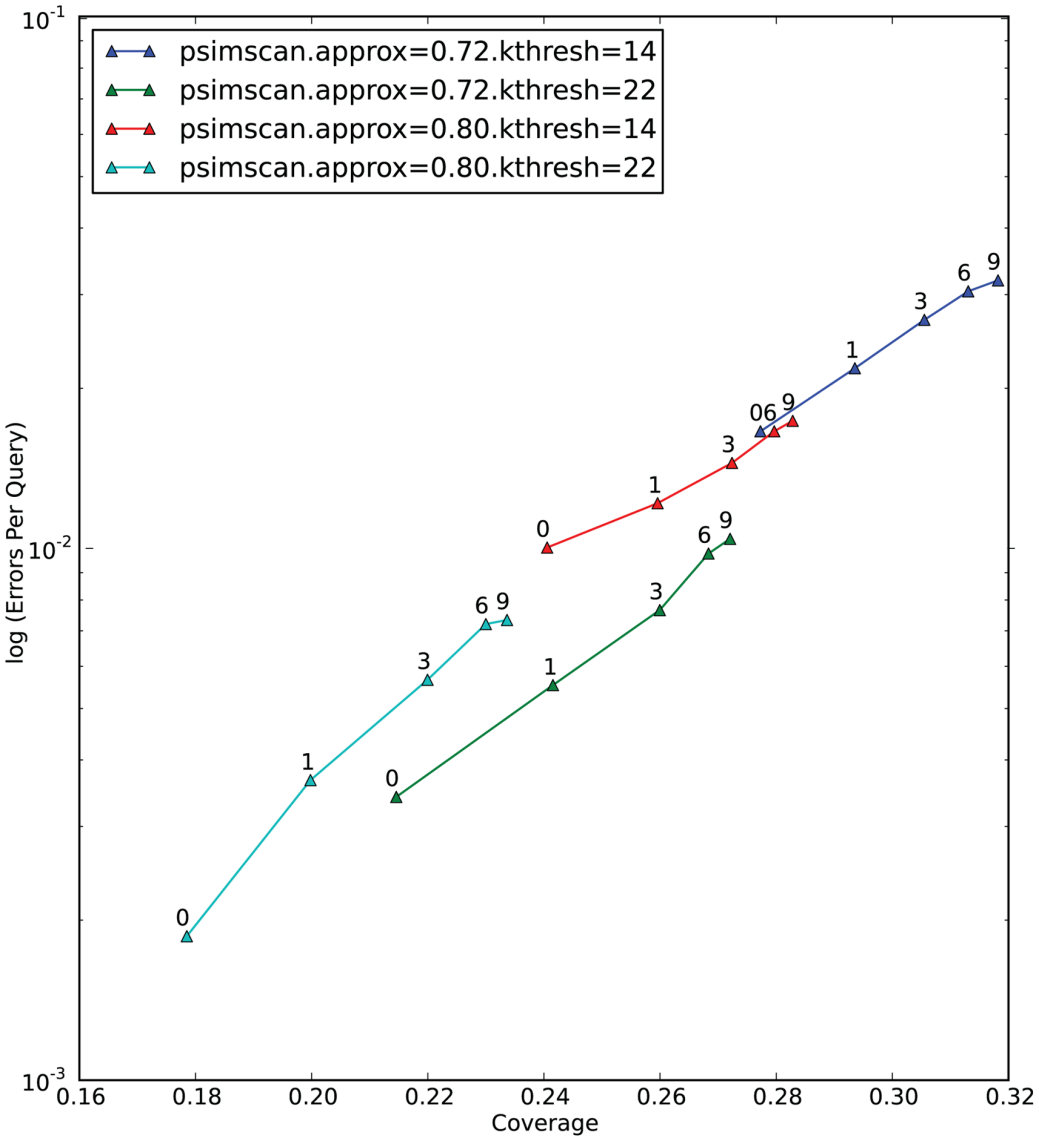
Selectivity and Sensitivity of PSimScan at different maximum diagonal shift values. Please see Fig. 5 for explanation of coordinate axes and method used. The dependency of Coverage and EPQ on the maximum diagonal shift taken at 4 different combinations of *approx*/*kthresh* values is shown. The dots on graphs are labeled with the values of *mxshift* parameter.

Our choice of those five tools was directed by the following considerations: NCBI BLASTP [2] still appears to be the most widely used tool for protein comparison, and to many researchers it remains a de-facto standard against which all other tools are evaluated. SSEARCH [4] can be seen as an ultimate tool to compute edit-distance based similarities not compromised by hit selection heuristics. USEARCH [33], while being closed-source and distributed under a restrictive license, in recent years has become an ubiquitously used tool due to its speed and accuracy. BLAT [18] has been a tool of choice in many studies due to its extreme speed. RapSEARCH [32] is an open-source tool designed specifically to increase the sensitivity of protein search while keeping it fast enough for analyzing transcriptomic and metagenomic datasets. To get comparable graphs for all tools, we recomputed E-values for each of detected similarities using SSEARCH. Fig. 5 presents dependency of EPQ and Coverage on *kthresh* and *approx* parameters of PSimScan. Fig. 6 presents dependency of EPQ and coverage on PSimScan’s *mxshift* parameter.

Presented results demonstrate that in selected parametric sub-space (for *kthresh* ≥18, especially for higher *approx* values) PSimScan shows better coverage-to-error rates than any of the evaluated standard tools. At *kthresh* = 26 and *approx* = 0.8 PSimScan reliably detects only relatively strong similarities: its coverage matches coverage by other tools at E-values below ∼1e–18. The error rate at these settings is 20 times lower than the error rate of RAPSearch for similarities with E-value below 1e–20, and about 2 times lower then error rates of BLAST, USEARCH or SSEARCH.

One can argue that the selectivity and sensitivity of the tools might be different when calculated on a balanced selection of sequences from different protein families rather than on the entire PDB90, where certain families are largely overweighed. We recreated Fig. 5 based on a subset of PDB90 with balanced representation of protein families. The resulting Fig. S1 (see Supplementary data) did not show any substantial difference from Fig. 5.

In our performance evaluation, we used E-values calculated by SSEARCH instead of those reported by the individual tools, for the following reason: SSEARCH is using a well-known standard algorithm of E-value calculation, whereas each tool calculates E-values differently, which makes the result of comparison unreliable. In the Supplementary data, we provide an alternative diagram for performance comparison which lists found and missed similarities by E-value, where E-values were calculated within the tools (Fig. S2). One notion that USEARCH did not find any similarities with E-values between 1e–60–1e–80 (which most probably means that these were classified differently) evidently proves that this alternative comparison method would be questionable at best.

#### Performance assessment with bacterial proteome vs. nr and uniprot benchmarks

While the ‘golden set’ benchmark described above allows to assess the quality of the results and find optimal parameters, it is not suitable for testing the computing time. The subject set in this test is so small that most of the run time is spent on the data pre-processing rather than the actual similarity search. In real-life usage scenarios, the subject databases are much larger. To perform the search speed testing, we used a comparison of the entire bacterial proteome of *S. pneumoniae* to standard protein databases: NCBI nr and UniProt/SwissProt. Along with PSimScan, we used NCBI BLAST, RAPSearch, USEARCH and BLAT for such testing. Out of these, only NCBI BLAST, PSimScan and RAPSearch were capable of processing NCBI “nr” database as the subject set on our test PC; both USEARCH and BLAT have run out of memory. However, all tools were capable of processing SwissProt/UniProt database which we have chosen for a more detailed comparison.

For the testing with the NCBI “nr” database, we used default values for all parameters, except for the PSimScan parameter ‘–rpq’ (maximal results per query), which was set to 8000; the same value was used for ‘-v’ parameters of NCBI BLAST and RAPSearch. The results of this testing are presented in Table 1:

**Table 1:** Performance testing against NCBI nr database.

Application	Run time	Speedup vs. BLAST
BLAST	66 hr 29 min	1×
PSimScan	6 hr 25 min	10.1×
RAPSearch	2 hr 37 min	32.3×
RAPSearch (including pre-processing)*	4 hr 04 min	16.3×

* Required pre-processing took 1 hr 27 min and created a 29.6 Gb index file.

**Figure 7:**
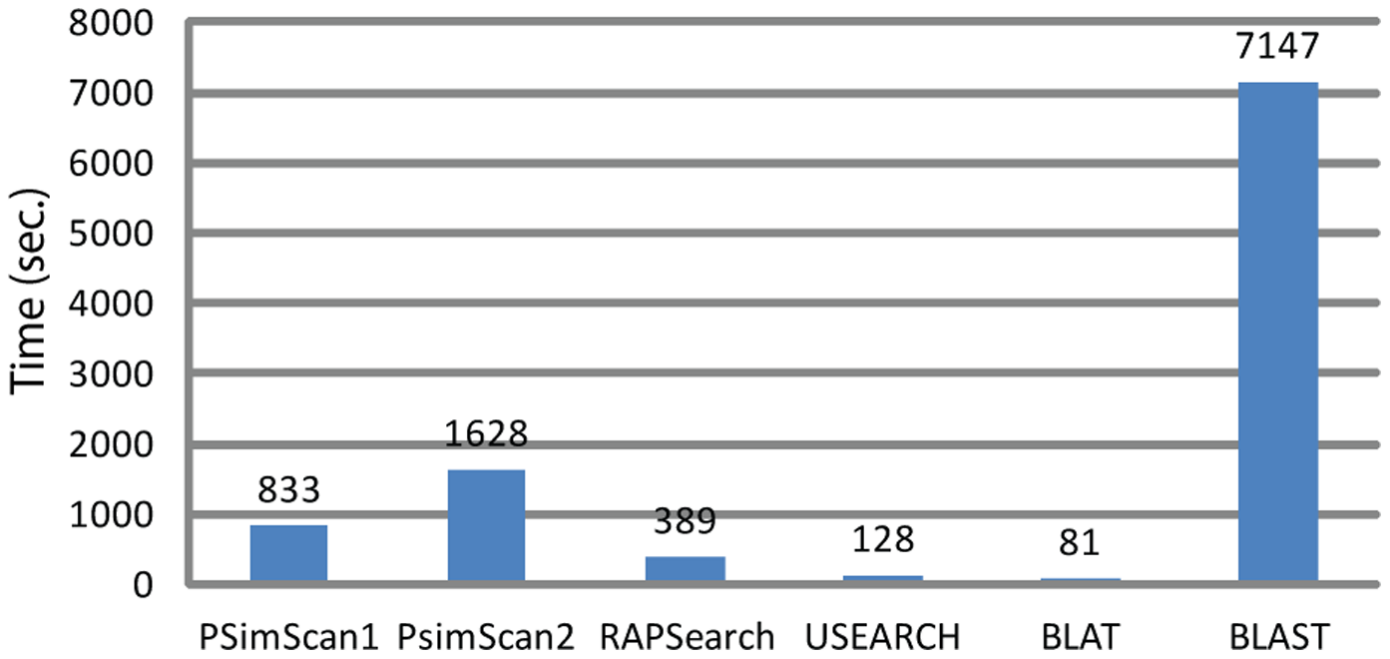
Processing speed for different quick protein similarity search tools. All measurements were taken at default parameters but for the “PSimScan2” series (*approx*: 0.79, *kthresh*: 14). *Streptococcus pneumoniae* R6 proteome was used as the query set, SwissProt/UniProt database – as the subject set.

A comparison of PSimScan compute time to those of RAPSearch, USEARCH, and BLAT with SwissProt/UniProt database as the subject set is shown on Fig. 7. For this test, PSimScan is shown twice, at default parameters (PSimScan1) and at *approx* (k-tuple diversification) 0.79 and *kthresh* (zone detection threshold) 14 (PSimScan2).

**Figure 8:**
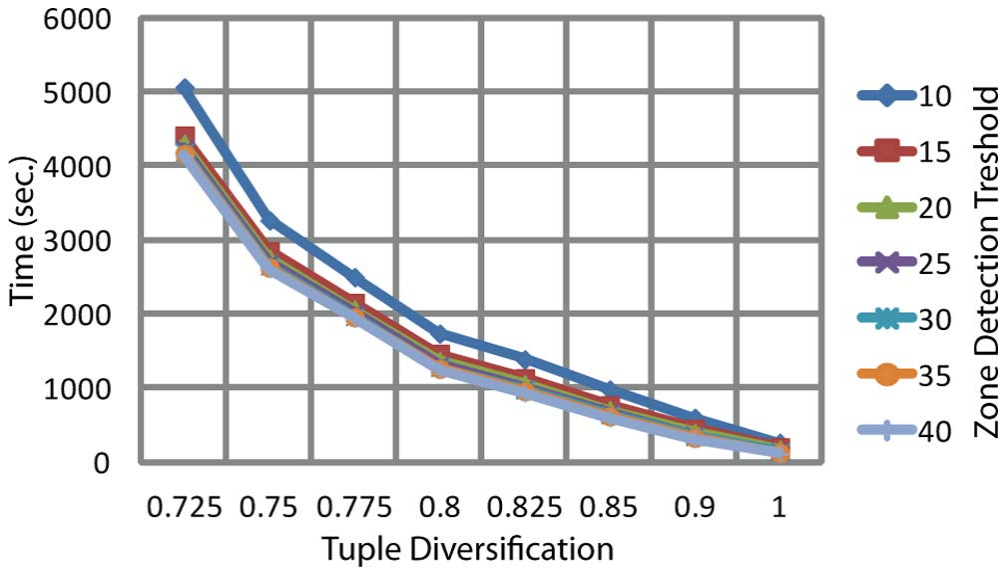
PSimScan processing time dependence on tuple diversification and similarity zone detection threshold. *Streptococcus pneumoniae* R6 proteome was used as the query set, SwissProt/Uniprot database – as the subject set. Measurements were taken at *mxshift* = 4.

**Figure 9:**
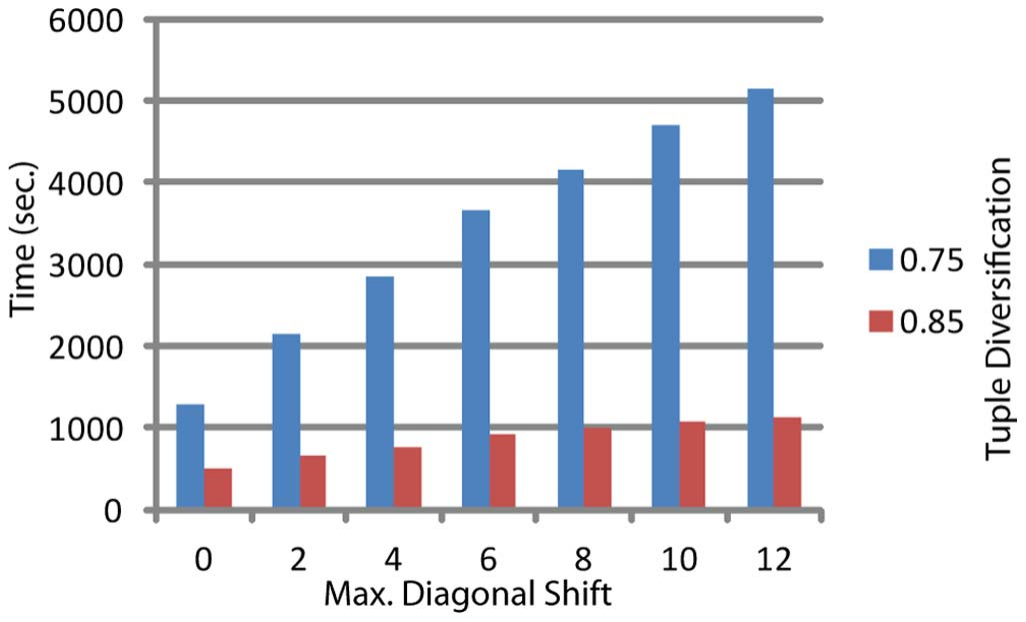
PSimScan processing time dependence on maximum diagonal shift. *Streptococcus pneumoniae* R6 proteome was used as the query set, SwissProt/Uniprot database – as the subject set. Measurements were taken at *kthresh*: 15 and *approx:* 0.75 and 0.85.

Dependency of compute time on the tuple diversification (*approx*) and zone detection threshold is shown on Fig. 8, and dependency on the number of adjusted diagonals looked up for a similarity zone expansion/merge is shown on Fig. 9.

### Conclusions

The above analysis demonstrates the flexibility of PSimScan which can be tuned to a particular task, from rapid detection of strong hits to a slow search for remote homologies. It also shows that PSimScan is best suited for the search of moderately strong similarities at E-values below 1e–30, where it outperforms BLAST (and SSEARCH) in speed and all described tools in coverage to errors ratio. The shown graphs also allow for choosing reasonable parameter settings for different usage scenarios. Tuple diversification (*approx* parameter) has the most impact on all performance characteristics (speed, sensitivity and selectivity). The maximum diagonal shift (*mxshift* parameter) increases sensitivity only up to the value of 3–4; above that, it just slows the execution. A lower zone detection threshold (*kthresh* parameter) can be used to increase the chance of finding longer and weaker similarities, and in general significantly correlates with specificity. The impact of the zone detection threshold (*kthresh*) on the execution time is steady but not very prominent.

In the above tests, PSimScan does not detect some of the weak similarities found by NCBI BLAST, because we used k-tuple size of 5, and BLAST by default uses k-tuple size of 3. Penta-peptide diversification at the levels above 0.7 cannot cover the difference in the initial hit detection stringency. Thus, BLAST finds some of the hits that just do not have matches long and strong enough to be seen in the PSimScan lookup. At k-tuple size of 3, PSimScan detects all BLAST similarities, however, PSimScan is not optimized for such operations and runs significantly slower then NCBI BLAST on short k-tuple sizes.

### Availability and Future Directions

Reference implementation for PSimScan is open source software available for download and usage under a GPL license at http://qsimscan.googlecode.com/files/qsimscan.tar.gz.

Besides open source tools, the PSimScan algorithm is used in several components of SciDM’s (scidm.org) EST processing pipeline: repeat search tool, contamination screening tool and massively parallel similarity matrix computing utility. They are implemented as network services controlled through a CORBA-based interface. The algorithm is also used in the Genome Designs (genomedesigns.com) genome annotation pipeline.

## Discussion

### The Importance of Fine Control

Careful algorithm design and code optimization with respect to the capabilities of the underlying computing system are equally important for saving computing and research time. However, it is even more important to choose the right method (and right parameters) for each particular task.

Computing similarities with high sensitivity takes much more resources than computing with low sensitivity, when only strong hits are detected. In many practical cases high-sensitivity search makes sense only for a fraction of sequences, namely those lacking strong similarities that would allow arranging them into families. For those that do associate, the use of consensuses and conserved motifs poses a much better utility for finding weak inter-family relationships than a direct comparison of individual members.

Therefore, an optimal strategy would be using a fast, rough comparison to assemble strong clusters, and then performing a slow and sensitive search for affiliations of sequences which do not easily associate. Incremental setups provide even more room for decreasing the computing complexity, as the initial search can be done against a relatively small set of ‘family representatives’.

It should be also noted that weak hits are usually hard to interpret, especially in automatic pipelines. Alternative methods based on motifs, statistical models or feature analyses are preferred for sequences lacking strong hits.

To be useful within this iterative approach, similarity search tools have to support a high degree of control over sensitivity-for-speed trading. Standard similarity search software tools have limited configurability. In PSimScan we aimed to overcome this limitation, allowing users to choose modes of operation best suitable for their particular tasks, from very fast and rough to more sensitive but slower searches.

### Query-based Lookup Table vs. Subject-based

For methods derived from the Pearson and Lipman search, the lookup tables can be constructed for query sequences (NCBI BLAST [2], Wublast [3], [32]), subject databases (BLAT [18]), or both. The usage of indexed subjects saves time otherwise spent on sequential reading of database records from disk. If the target architecture has enough fast-access memory to hold an index for the entire subject database, time saving will be proportional to the disk read time. When many searches are performed against a standard subject database(s) like in the NCBI public service setup, such indexing can provide a tremendous increase in speed. Unfortunately, consumer-grade computer systems and even standard high-performance servers typically do not have such RAM capacity; hence the subject indexing incurs a considerable overhead of custom setups like distributing search tasks across multiple CPUs, each handling a portion of a database. For searches against specific subsets, these subsets should be either independently pre-indexed, or require a post-search result filtering, which would result in unnecessary consumption of CPU time.

On the other hand, query indexing requires a much simpler memory management and allows to search against specific subsets in a database by loading only desired subject sequences. Additional time spent on the query set indexing is neglectful for smaller tasks. For the bulky jobs like searching the entire NR against itself, both indexing methods are equivalently heavy, since the entire database gets tuple-indexed in either case.

The three popular tools for fast search mentioned above use some form of subject indexing: USEARCH and BLAT construct an array of word counts and a lookup table, respectively, on the fly, while RAPSearch uses a pre-build suffix array-based index. The implementation of USEARCH and BLAT makes it difficult to run searches in large subject sets on conventional hardware: 8 Gb RAM appears to be insufficient for search against the NCBI ‘nr’, as well as in a typical metagenomic data set. Using these tools for such databases requires special hardware or additional configuration efforts. RAPSearch does not have such limitations; however, it still requires pre-building of an index (which takes time, though amortized in multiple searches) as well as additional maintenance.

In our tool, we use query indexing to allow execution on the low-end consumer-grade machines, and to provide users with the flexibility of searching against desired subsets of databases without the overhead of lookup table re-creation and maintenance.

### Direct Lookup Versus Tree-based Dictionary Search

The inner loop of PSimScan, similarly to other algorithms based on the dictionary lookup, consists of reading the next tuple from the subject sequence and searching for it in the dictionary. Since the search is performed for every position in the sequence, the timing of the entire search is proportional to the timing of an individual search event. Thus, it is important that the search events have constant-bound time. Natural arrangement of tuples in a tree-based structure or in an ordered array would impose a logarithmic search time. In order to keep the tuple search time constant, we used direct addressing of the Lookup Table, where binary representation of a tuple serves as an address (offset) in the table. To reduce the overhead of multiple dynamic conversions, the sequences are internally represented in a form that minimizes operation of fragment-to-offset translation.

It must be pointed out that in cases when pre-processing of the query sequences is possible, representing indexes as ordered lists of actually occurring words (of fixed or variable length) creates an opportunity for additional optimization through pre-sorting batches of words derived from queries, and merge-walking query and suffix ‘indices’ simultaneously. Using this approach for finding inexact matches would be more complicated. We do not use it in the current implementation of PSimScan, but may consider it in future versions.

### Use of Similarity Zones Versus Hit Extension

Sequence alignment and Karlin-Altschul statistics are both computationally-intense tasks. Delaying these to later processing stages, where fewer candidates remain after filtering, saves computing time. In PSimScan, the alignments are computed only in the rectangular ‘similarity zones’ on the diagonal matrix, where primary match concentration is significantly high. Detection of such zones is much quicker than building alignments on all the zone’s matches. This approach allows for skipping k-tuple matches that do not belong to dense enough zones. It also provides an advantage over diagonal-based score accumulation, since the latter does not account for short gaps and therefore has more limited sensitivity.

One of the aforementioned tools for quick protein similarity search, BLAT, uses a similar approach, counting matches that appear on neighboring diagonals. It first accumulates all matches, then sorts them in the order of increasing diagonal index, and afterwards uses a sliding window to count hits on the sorted array of matches. This essentially defers similarity zone detection to the time when all hits have been seen. In PSimScan we build similarity zones dynamically, looking up the adjacent diagonals for each hit. Thus, time is not spent on sorting, but on the diagonal lookup, which is more efficient on the small neighborhoods of up to 3–4 adjacent diagonals.

Limiting the width of the diagonal lookup zone implies that found similarity zones would not extend over long gaps. To compensate for that, we merge zones that appear to be close enough to form a good alignment. The procedure for such merging is very efficient, with the run time proportional to the number of properly ordered zone pairs, which is small for typical similarities.

### Use of Banded Alignment

Accurate alignment is preferable while calculating scores and presenting results; however, classic alignment approaches depend on computing rectangular traceback matrices, which is slow (O(nm) bound) and space-consuming. It is also totally impractical when longer sequences are compared, because of m*n space requirements. Early detection of similarity zones makes it possible to compute tracebacks on parts of the alignment space, assuming that the likelihood of an alignment running out of such zone is low. Such ‘banded’ alignment significantly saves both space and time, in most cases reducing m*n complexity to a much lesser zone_width*zone_length (zone_width is usually small).

### Using Weighted k-tuples

As different k-tuples occur in sequences with different frequencies, the importance of each particular match depends on the k-tuple sequence: a match with an ubiquitous k-tuple is less important than with a unique one. PSimScan can utilize a table of relative statistical weights of different tuples computed via external means by comparing frequencies of tuple occurrences in real sequence databases. It seems natural to exploit relative frequencies computed on sequence sets used in real similarity searches, though it is impossible when the sets are small. Pre-computing arrays of frequencies on the entire domains of GenBank also proved to be helpful. Working with relative frequencies clipped to pre-defined boundaries usually improves search results.

The use of tuple frequencies gives a visible sensitivity burst for extremely weak hits with no significant effect on strong matches. A software tool for collecting tuple occurrence statistics is easy to devise; it is also available by request from the authors of this publication.

### Post-processing of Zone Alignments

In many practical cases, for example, with multi-domain or fusion proteins, a pair of sequences can carry several remote segments of relatively strong similarities separated by extended dissimilar zones. Popular tools, including BLAST, RAPSearch, USEARCH, and BLAT, treat and report such similarities as separate. In certain cases it is convenient to merge them into a super-alignment using a globally optimal arrangement of segments and gaps. PSimScan adds an optional post-processing stage for such merges.

Another frequent case of multiple similarity segments appearing in a sequence pair is when either (or both) of them contain repeats. PSimScan provides an option to filter all similarities caused by repeated segments, leaving only the strongest one. This filter operates on any arrangement of repeats, and can be combined with remote segment merger.

### Conclusion

PSimScan utility provides users with an option to perform similarity search significantly faster than using NCBI BLAST, with no need to invest in computer infrastructure beyond a standard PC. The underlying algorithm allows flexible speed-for-sensitivity trading by altering search parameters. This is especially useful in many practical cases where a fast detection of relatively strong similarities is desirable.

We see PSimScan as especially useful in iterative high-throughput systems, where the majority of strong similarities can be detected very quickly, and then the remaining unclear relationships have to be evaluated by slower but more sensitive methods such as profile, position-specific matrix- or HMM-based ones.

## Supporting Information

Figure S1 **Selectivity and Sensitivity of PSimScan at different parameters versus other similarity search tools, calculated on a normalized database.** All proteins from a subset of the PDB90 database with balanced representation of protein families were compared with each other using PSimScan, SSEARCH, BLAST, USEARCH, RAPSearch and BLAT. PSimScan was tested at different combinations of *kthresh* (similarity zone detection threshold) and *approx* (tuple diversification level) parameters. For SSEARCH, BLAST, USEARCH, RAPSearch and BLAT, the Coverage vs Error graphs were plotted as described by Brenner *et al* [47]. Similarities between proteins of the same SCOP fold were treated as true positives, while similarities between proteins of different folds – as false positives (errors). The Coverage is the ratio between the number of true positives and the total number of protein pairs, where both members belong to the same fold. The EPQ is the ratio between the number of detected false positives and the number of queries. The Coverage-vs.-Error graph contains points in Coverage/EPQ plane which correspond to the sets of similarities with E-values below a given cut-off (some dots on the graphs are labeled with E-values). To get comparable graphs for different tools, we re-computed the E-values for all detected similarities with SSEARCH, and used those E-values for the graph construction. We ran PSimScan at all combinations of 6 different *kthresh* values (shown in legend) and 7 different *approx* values. For each run, total coverage and EPQ were computed and plotted. On each curve corresponding to a particular *kthresh*, the triangles mark the following *approx* values, left to right: 1.0, 0.95, 0.9, 0.85, 0.8, 0.76, 0.72.
(TIF)

Figure S2 **Performance comparison for quick protein similarity search tools calculated using the tools’ own reporting functionality.** All measurements were taken at default parameters but for the “PSimScan2” series (‘approx’: 0.79, ‘kthresh’: 14). *Streptococcus pneumoniae* R6 proteome was used as the query set, SwissProt/Uniprot database – as the subject set. A. Found similarities by E-value (‘according to the tools’ own reporting - here and below). B. % of missed similarities compared to NCBI BLAST, by E-value.
(TIF)

## References

[1] Altschul SF, Gish W, Miller W, Myers EW, Lipman DJ (1990) Basic local alignment search tool. Journal of molecular biology, 215 (3): 403–410. 10.1016/S0022-2836(05)80360-2 2231712

[2] Altschul SF, Madden TL, Schaffer AA, Zhang J, Zhang Z, et al (1997) Gapped BLAST and PSI-BLAST: a new generation of protein database search programs. Nucleic acids research, 25 (17): 3389–3402. 10.1093/nar/25.17.3389 PMC146917 9254694

[3] Gish W (1996–2009) Advanced Biocomputing website. Available: http://blast.advbiocomp.com. Accessed 2013 Feb.7.

[4] Pearson WR, Lipman DJ (1988) Improved tools for biological sequence comparison. Proceedings of the National Academy of Sciences of the United States of America, 85 (8): 2444–2448. 10.1073/pnas.85.8.2444 PMC280013 3162770

[5] Pearson WR (1990) Rapid and sensitive sequence comparison with FASTP and FASTA. Methods in enzymology 183: 63–98. 2156132 10.1016/0076-6879(90)83007-v

[6] Pearson WR (2000) Flexible sequence similarity searching with the FASTA3 program package. Methods Mol Biol 132: 185–219. 10547837 10.1385/1-59259-192-2:185

[7] Biegert A, Soding J (2009) Sequence context-specific profiles for homology searching. Proceedings of the National Academy of Sciences of the United States of America, 106 (10): 3770–3775. 10.1073/pnas.0810767106 PMC2645910 19234132

[8] Merkeev IV, Mironov AA (2006) PHOG-BLAST–a new generation tool for fast similarity search of protein families. BMC evolutionary biology 6: 51. 16792802 10.1186/1471-2148-6-51 PMC1522020

[9] Zhang Z, Schaffer AA, Miller W, Madden TL, Lipman DJ, et al (1998) Protein sequence similarity searches using patterns as seeds. Nucleic acids research, 26 (17): 3986–3990. 10.1093/nar/26.17.3986 PMC147803 9705509

[10] Eddy SR (1998) Profile hidden Markov models. Bioinformatics, 14 (9): 755–763. 10.1093/bioinformatics/14.9.755 9918945

[11] Eddy SR (2009) A new generation of homology search tools based on probabilistic inference. Genome informatics International Conference on Genome Informatics, 23 (1): 205–211. 20180275

[12] Johnson LS, Eddy SR, Portugaly E (2010) Hidden Markov model speed heuristic and iterative HMM search procedure. BMC bioinformatics 11: 431. 20718988 10.1186/1471-2105-11-431 PMC2931519

[13] Eddy SR (2011) Accelerated Profile HMM Searches. PLoS computational biology, 7 (10): e1002195. 10.1371/journal.pcbi.1002195 PMC3197634 22039361

[14] Cameron M, Williams HE, Cannane A (2006) A deterministic finite automaton for faster protein hit detection in BLAST. Journal of computational biology : a journal of computational molecular cell biology, 13 (4): 965–978. 10.1089/cmb.2006.13.965 16761921

[15] Cameron M, Williams HE, Cannane A (2004) Improved gapped alignment in BLAST. IEEE/ACM transactions on computational biology and bioinformatics/IEEE, ACM, 1 (3): 116–129. 10.1109/TCBB.2004.32 17048387

[16] Cameron M, Williams HE (2007) Comparing compressed sequences for faster nucleotide BLAST searches. IEEE/ACM transactions on computational biology and bioinformatics/IEEE, ACM, 4 (3): 349–364. 10.1109/TCBB.2007.1029 17666756

[17] Hughey R, Krogh A (1995) SAM: Sequence alignment and modeling software system. UCSC Bioinformatics (Computational Biology) website. Available: http://compbio.soe.ucsc.edu/sam.html. Accessed 2013 Feb. 7.

[18] Kent WJ (2002) BLAT–the BLAST-like alignment tool. Genome research, 12 (4): 656–664. 10.1101/gr.229202 PMC187518 11932250

[19] Rognes T (2001) ParAlign: a parallel sequence alignment algorithm for rapid and sensitive database searches. Nucleic acids research, 29 (7): 1647–1652. 10.1093/nar/29.7.1647 PMC31274 11266569

[20] Saebo PE, Andersen SM, Myrseth J, Laerdahl JK, Rognes T (2005) PARALIGN: rapid and sensitive sequence similarity searches powered by parallel computing technology. Nucleic acids research, 33 (Web Server issue): W535–539. 10.1093/nar/gki423 PMC1160184 15980529

[21] Cameron M, Bernstein Y, Williams HE (2007) Clustered sequence representation for fast homology search. Journal of computational biology : a journal of computational molecular cell biology, 14 (5): 594–614. 10.1089/cmb.2007.R005 17683263

[22] Worley KC, Wiese BA, Smith RF (1995) BEAUTY: an enhanced BLAST-based search tool that integrates multiple biological information resources into sequence similarity search results. Genome research, 5 (2): 173–184. 10.1101/gr.5.2.173 9132271

[23] Gouveia-Oliveira R, Sackett PW, Pedersen AG (2007) MaxAlign: maximizing usable data in an alignment. BMC bioinformatics 8: 312. 17725821 10.1186/1471-2105-8-312 PMC2000915

[24] Esteban DJ, Syed A, Upton C (2007) Organizing and updating whole genome BLAST searches with ReHAB. Methods Mol Biol 395: 187–194. 17993674 10.1007/978-1-59745-514-5_11

[25] Wang C, Lefkowitz EJ (2004) SS-Wrapper: a package of wrapper applications for similarity searches on Linux clusters. BMC bioinformatics 5: 171. 15511296 10.1186/1471-2105-5-171 PMC545957

[26] Hochreiter S, Heusel M, Obermayer K (2007) Fast model-based protein homology detection without alignment. Bioinformatics, 23 (14): 1728–1736. 10.1093/bioinformatics/btm247 17488755

[27] Rajasekaran S, Nick H, Pardalos PM, Sahni S, Shaw G (2001) Efficient Algorithms For Local Alignment Search. Journal of Combinatorial Optimization, 5(1) 2001: 117–124.

[28] Agrawal R, Faloutsos C, Swami A (1993) Efficient similarity search in sequence databases. FOUNDATIONS OF DATA ORGANIZATION AND ALGORITHMS, Lecture Notes in Computer Science, 730/1993 (69–84).

[29] Rajasekaran S, Jin X, Spouge JL (2002) The efficient computation of position-specific match scores with the fast fourier transform. Journal of computational biology : a journal of computational molecular cell biology, 9 (1): 23–33. 10.1089/10665270252833172 11911793

[30] Li H, Homer N (2010) A survey of sequence alignment algorithms for next-generation sequencing. Briefings in bioinformatics, 11 (5): 473–483. 10.1093/bib/bbq015 PMC2943993 20460430

[31] Kahn SD (2011) On the future of genomic data. Science, 331 (6018): 728–729. 10.1126/science.1197891 21311016

[32] Baker M (2010) Next-generation sequencing: adjusting to data overload. Nat Meth 7, 495–499.

[33] Edgar RC (2010) Search and clustering orders of magnitude faster than BLAST. Bioinformatics, 26 (19): 2460–2461. 10.1093/bioinformatics/btq461 20709691

[34] Ye Y, Choi JH, Tang H (2011) RAPSearch: a fast protein similarity search tool for short reads. BMC bioinformatics 12: 159. 21575167 10.1186/1471-2105-12-159 PMC3113943

[35] Zhao Y, Tang H, Ye Y (2012) RAPSearch2: a fast and memory-efficient protein similarity search tool for next-generation sequencing data. Bioinformatics, 28 (1): 125–126. 10.1093/bioinformatics/btr595 PMC3244761 22039206

[36] Lipman DJ, Pearson WR (1985) Rapid and sensitive protein similarity searches. Science, 227 (4693): 1435–1441. 10.1126/science.2983426 2983426

[37] Dayhoff MO, Schwartz RM, Orcutt BC (1978) Atlas of Protein Sequence and Structure. Dayhoff MO, editor vol. 5. Suppl. 3 (Washington, DC: National Biomedical Research Foundation): p. 345–352.

[38] Dayhoff MO, Schwartz RM, Orcutt BC (1978) Atlas of Protein Sequence and Structure. Dayhoff MO, editor, vol. 5. Suppl. 3 (Washington, DC: National Biomedical Research Foundation): 353–358.

[39] Altschul SF (1993) A protein alignment scoring system sensitive at all evolutionary distances. Journal of molecular evolution, 36 (3): 290–300. 10.1007/BF00160485 8483166

[40] Henikoff S, Henikoff JG (1992) Amino acid substitution matrices from protein blocks. Proceedings of the National Academy of Sciences of the United States of America, 89 (22): 10915–10919. 10.1073/pnas.89.22.10915 PMC50453 1438297

[41] Sedgewick R, Wayne K (2011) Algorithms, 4th edn: Addison-Wesley Professional.

[42] Altschul SF (1998) Generalized affine gap costs for protein sequence alignment. Proteins, 32 (1): 88–96. 9672045

[43] Needleman SB, Wunsch CD (1970) A general method applicable to the search for similarities in the amino acid sequence of two proteins. Journal of molecular biology, 48 (3): 443–453. 10.1016/0022-2836(70)90057-4 5420325

[44] Smith TF, Waterman MS (1981) Identification of common molecular subsequences. Journal of molecular biology, 147 (1): 195–197. 10.1016/0022-2836(81)90087-5 7265238

[45] Karlin S, Altschul SF (1990) Methods for assessing the statistical significance of molecular sequence features by using general scoring schemes. Proceedings of the National Academy of Sciences of the United States of America, 87 (6): 2264–2268. 10.1073/pnas.87.6.2264 PMC53667 2315319

[46] Mott R (2000) Accurate formula for P-values of gapped local sequence and profile alignments. Journal of molecular biology, 300 (3): 649–659. 10.1006/jmbi.2000.3875 10884359

[47] Brenner SE, Chothia C, Hubbard TJ (1998) Assessing sequence comparison methods with reliable structurally identified distant evolutionary relationships. Proceedings of the National Academy of Sciences of the United States of America, 95 (11): 6073–6078. 10.1073/pnas.95.11.6073 PMC27587 9600919

[48] Chandonia JM, Hon G, Walker NS, Lo Conte L, Koehl P, et al.. (2004) The ASTRAL Compendium in 2004. Nucleic acids research, 32 (Database issue): D189–192. 10.1093/nar/gkh034 PMC308768 14681391

