# Supplementary figures and images for: PSimScan: Algorithm and Utility for Fast Protein Similarity Search
Source: PLoS One. 2013 Mar 7;8(3):e58505. doi: 10.1371/journal.pone.0058505 (PMC3591303; doi:10.1371/journal.pone.0058505)

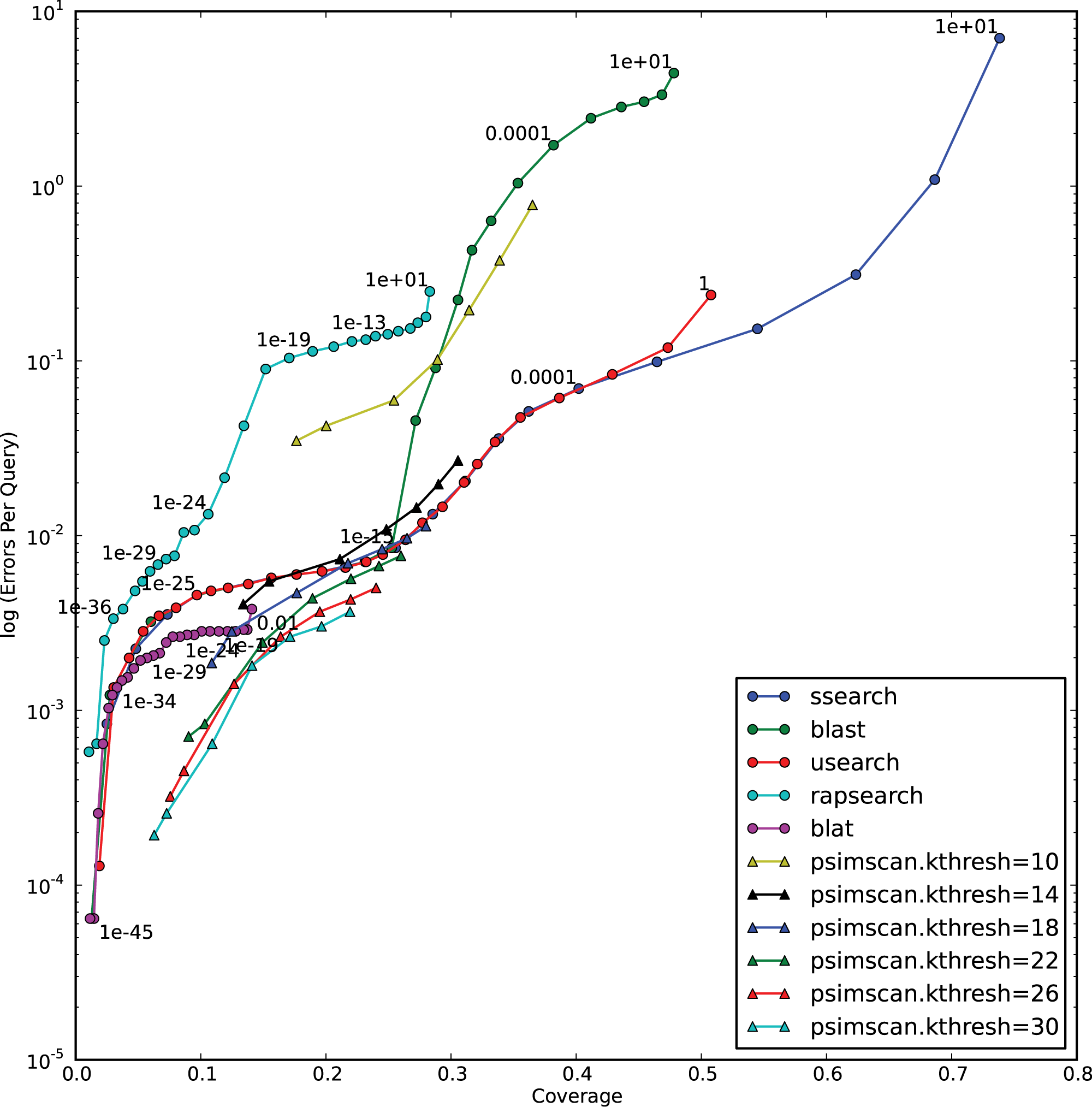

Supplement: Figure S1 — Selectivity and Sensitivity of PSimScan at different parameters versus other similarity search tools, calculated on a normalized database. All proteins from a subset of the PDB90 database with balanced representation of protein families were compared with each other using PSimScan, SSEARCH, BLAST, USEARCH, RAPSearch and BLAT. PSimScan was tested at different combinations of kthresh (similarity zone detection threshold) and approx (tuple diversification level) parameters. For SSEARCH, BLAST, USEARCH, RAPSearch and BLAT, the Coverage vs Error graphs were plotted as described by Brenner et al [47]. Similarities between proteins of the same SCOP fold were treated as true positives, while similarities between proteins of different folds – as false positives (errors). The Coverage is the ratio between the number of true positives and the total number of protein pairs, where both members belong to the same fold. The EPQ is the ratio between the number of detected false positives and the number of queries. The Coverage-vs.-Error graph contains points in Coverage/EPQ plane which correspond to the sets of similarities with E-values below a given cut-off (some dots on the graphs are labeled with E-values). To get comparable graphs for different tools, we re-computed the E-values for all detected similarities with SSEARCH, and used those E-values for the graph construction. We ran PSimScan at all combinations of 6 different kthresh values (shown in legend) and 7 different approx values. For each run, total coverage and EPQ were computed and plotted. On each curve corresponding to a particular kthresh, the triangles mark the following approx values, left to right: 1.0, 0.95, 0.9, 0.85, 0.8, 0.76, 0.72. (TIF) [file pone.0058505.s001.tif]

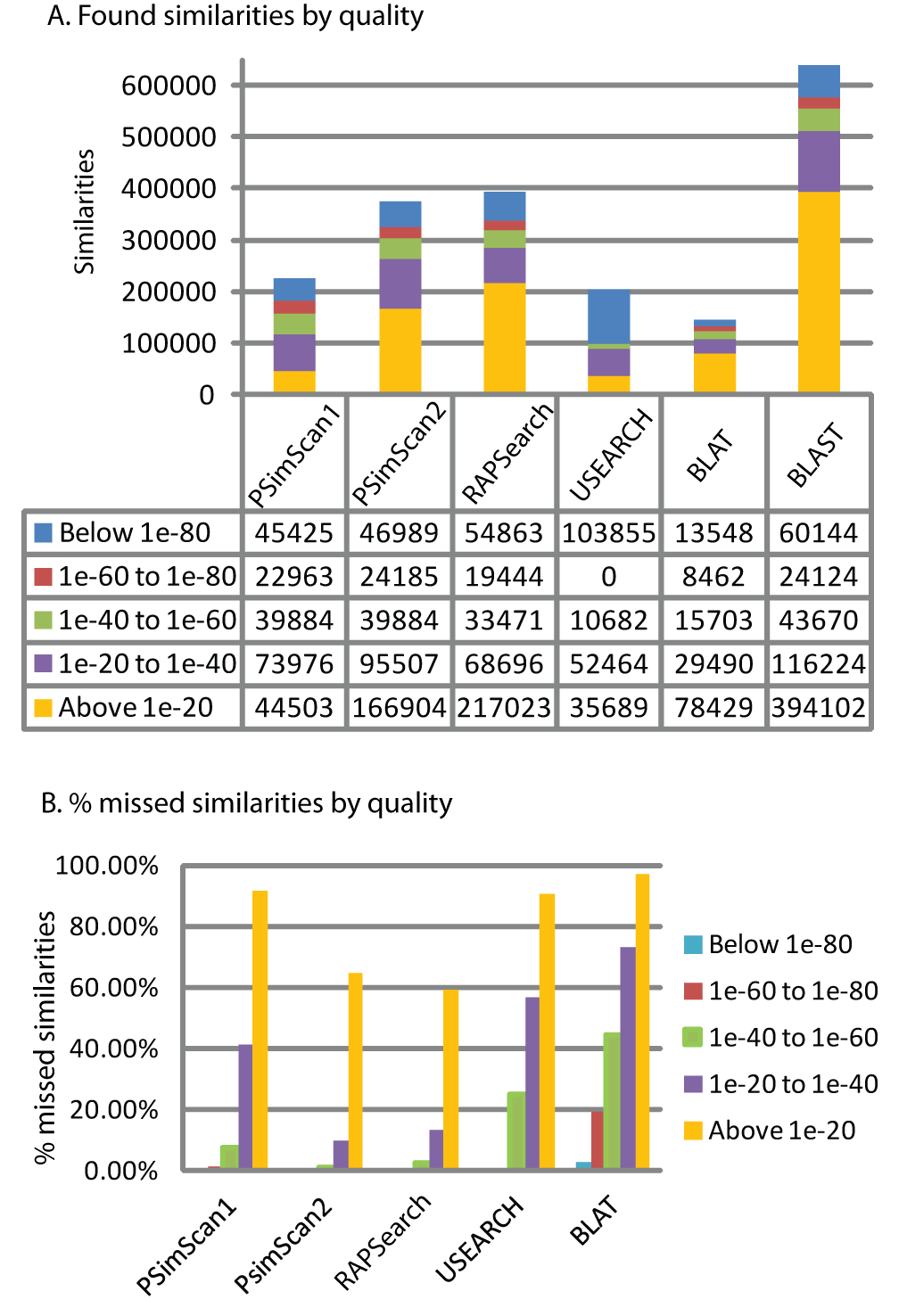

Supplement: Figure S2 — Performance comparison for quick protein similarity search tools calculated using the tools’ own reporting functionality. All measurements were taken at default parameters but for the “PSimScan2” series (‘approx’: 0.79, ‘kthresh’: 14). Streptococcus pneumoniae R6 proteome was used as the query set, SwissProt/Uniprot database – as the subject set. A. Found similarities by E-value (‘according to the tools’ own reporting - here and below). B. % of missed similarities compared to NCBI BLAST, by E-value. (TIF) [file pone.0058505.s002.tif]
